# An Aspergillus luchuensis isolated from a patient with hemoptysis insights from a comprehensive genome-based analysis: Case report

**DOI:** 10.1097/MD.0000000000050486

**Published:** 2026-08-28

**Authors:** Kai Chang, Huajie Xie, Yanyan Wang, Xin Zhao, Wanlin Na, Ningwei Xian, Yuan Liu, Zhongyong Jiang, Chenxia Liu

**Affiliations:** a Department of Laboratory Medicine, The General Hospital of Western Theater Command, Chengdu, Sichuan, China; b College of Medicine, Southwest Jiaotong University, Chengdu, Sichuan, China; c Department of medical Laboratory, Affiliated Cancer Hospital of Chengdu Medical College, Chengdu Seventh People’s Hospital, Chengdu, Sichuan, China; d Department of Infectious Diseases, The General Hospital of Western Theater Command, Chengdu, Sichuan, China; e Institute of Microbiology, Sichuan Center for Disease Control and Prevention, Chengdu, Sichuan, China.

**Keywords:** *Aspergillus luchuensis*, case report, invasive pulmonary aspergillosis, non-neutropenic, whole genome

## Abstract

**Rationale:**

*Asp luchuensis*, a member of the *A niger* group, is widely used in food fermentation and rarely causes invasive pulmonary aspergillosis (IPA) in humans. Clinical cases of IPA induced by this strain are extremely scarce, and its genomic characteristics, virulence profiles, and pathogenic mechanisms remain poorly understood, resulting in insufficient clinical recognition of its invasive infection potential.

**Patient concerns:**

A 57-year-old immunocompetent non-neutropenic male patient with a long-term smoking and drinking history presented with unexplained severe cough and massive hemoptysis (approximately100 mL) without other typical infectious symptoms.

**Diagnoses:**

Combined with chest computed tomography (CT) inflammatory lesions, positive galactomannan test, fungal PCR and metagenomic next-generation sequencing results, the patient was definitively diagnosed with probable *A luchuensis*-induced IPA. Genomic and transcriptomic analyses confirmed the pathogen as a variant *A luchuensis* strain with 3 key hypervirulence genes, highly active mitochondrial energy metabolism, and no specific antifungal resistance genes.

**Interventions:**

The patient received standardized intravenous antifungal combination therapy with voriconazole and amphotericin B after confirmed diagnosis.

**Outcomes:**

The patient’s cough and hemoptysis were significantly relieved after 10 days of treatment, with stable vital signs and no adverse drug reactions or disease progression.

**Lessons:**

*A luchuensis* possesses strong invasive pathogenicity and can trigger IPA even in non-neutropenic immunocompetent individuals. Negative conventional microbial tests cannot exclude its infection, and mNGS is a reliable diagnostic tool. This strain is susceptible to routine antifungal drugs, and clinicians should raise awareness of atypical *Asp* species-induced invasive pulmonary infections.

## 1. Introduction

The *Asp* genus is the pathogenic agent responsible for invasive aspergillosis (IA), a disease associated with high morbidity and mortality rates.^[1]^ Although *A fumigatus* remains the most common species within this genus, other *Asp* species, such as *A niger*, have been increasingly identified in recent years as causes of invasive diseases.^[2]^ *A niger* is widely distributed in natural environments and is also utilized in industrial production for the manufacture of pharmaceuticals, food ingredients, and enzyme preparations.^[3]^ *A luchuensis*, a member of the *A niger* group, is extensively used in food fermentation in East Asia, for example, in Korean meju and nuruk, Japanese awamori, and Chinese Puerh tea.^[4]^ This species is often associated with fungal ear infections, but has previously been rarely reported to cause invasive aspergillosis.^[5]^ Here, we report the first whole-genome and transcriptomic analyses of strains isolated from probable IPA caused by *A luchuensis*.

## 2. Case presentation

### 2.1. Medical history and symptoms

**Figure 1.**
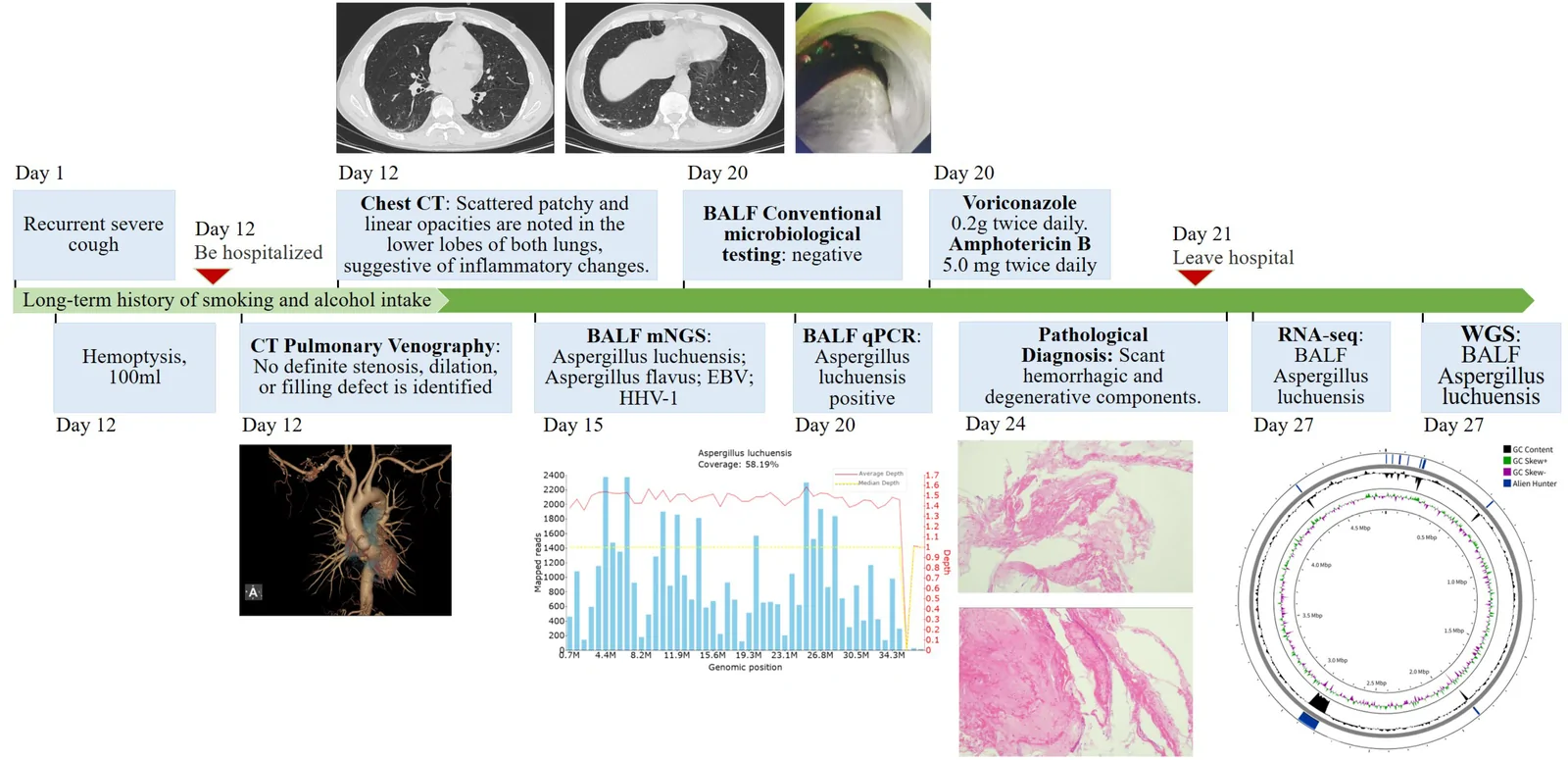
The patient’s entire diagnosis and treatment process. BALF = bronchoalveolar lavage fluid, CT = computed tomography.

A 57-year-old male patient, previously healthy, with a long-term history of smoking and alcohol consumption. The patient repeatedly experienced hemoptysis following severe coughing, presenting as bright red blood, with an approximate volume of 100mL. He was admitted to the hospital more than 10 hours after the hemoptysis episode. Physical examination upon admission: Conscious, responsive, with coherent answers. Bilateral pupils equal and approximately 0.3 cm, responsive to light. Heart rhythm regular, no significant murmurs heard over any cardiac valve areas. Chest wall without deformity; bilateral breath sounds coarse, no significant dry or wet rales heard. Abdomen soft, no tenderness, rebound tenderness, or muscle guarding. Murphy’s sign negative. No percussion pain in the liver and kidney regions. No shifting dullness. Bowel sounds normal. No edema in both lower limbs. The patient’s entire diagnosis and treatment process is illustrated in Figure 1.

### 2.2. Routine and radiographic examinations

On examination, White Blood Cell Count: 7.38 × 10^9^/L (normal value (3.5–9.5) × 10^9^/L), Hemoglobin concentration: 112 g/L (normal value (130–175) g/L), Platelet count: 95 × 10^9^/L (normal value (125–350) × 10^9^/L), Neutrophil percentage: 79.3% (normal value (40–75) %), Lymphocyte percentage: 16.5% (normal value (2050) %). T lymphocyte subsets (by flow cytometry): CD4+/CD8+ ratio: 3.37 (normal value (0.78–1.89)), CD4+ T lymphocyte percentage: 57.51% (normal value (24.93–45.57) %). The following tests showed no significant abnormalities: Glucose measurement, lipid profile, procalcitonin test, cardiac markers, thromboelastography, tumor markers, autoimmune antibodies, T-cell interferon-gamma release assay for tuberculosis infection, urinalysis, and fecal occult blood test.

Diagnosis of chest Computed tomography (CT): Bilateral pulmonary emphysematous changes; increased lung markings in both lungs. Scattered patchy and linear opacities are seen in the lingular segment of the left upper lobe, the medial segment of the right middle lobe, and both lower lobes, likely representing inflammatory and chronic fibrotic changes. CT Pulmonary Venography demonstrates normal origin of the pulmonary arteries and veins. The main pulmonary arteries and veins, along with their major branches, course and distribute normally, with no definite stenosis, dilation, or filling defects. Bilateral pulmonary emphysema with multiple bullae; scattered linear, patchy, and ground-glass opacities in both lungs, possibly indicative of inflammatory lesions. Small nodules in the lateral basal segment of the left lower lobe and the lingular segment of the left upper lobe, likely representing inflammatory or proliferative foci.

### 2.3. The examine of pathogeny

Auxiliary examination: No pathogenic growth was observed in the lavage fluid through routine bacterial smear, fungal smear, acid-fast bacillus (AFB) smear, fungal fluorescence staining, Mycobacterium tuberculosis DNA fluorescence PCR testing, as well as bacterial and fungal cultures bronchoalveolar lavage fluid Pathogenic Metagenomic Detection: *Asp* luchuensis (Sequence Reads: 20,318), *Asp* flavus (Sequence Reads: 83), Human Herpesvirus 4 (Sequence Reads: 38), and Human Herpesvirus 1 (Sequence Reads: 5). bronchoalveolar lavage fluid (1,3)-β-D-glucan test (G): positive and Galactomannan Detection (GM): positive; To confirm the presence of *Asp*, quantitative real-time PCR (qPCR) was performed to detect both genomic DNA. Results demonstrated genomic DNA positivity for *Asp*.

### 2.4. Diagnostic and therapeutic process

According to the criteria of European Organisation for Research and Treatment of Cancer/Mycoses Study Group (EORTC/MSG) and the Chinese “Guidelines for Diagnosis and Treatment of Invasive Pulmonary Fungal Disease,” based on risk factors (long-term residence in a humid environment, engagement in horticultural work), clinical features (cough, hemoptysis, nodular shadows on CT imaging), microbiological findings (positive GM test, PCR, and NGS for fungi), and histopathological results, the diagnostic level of this case is “probable.”

After diagnosis (Day 20), the patient received intravenous infusion of voriconazole and amphotericin B. The voriconazole was prescribed at an initial dose of 0.33 g/day, and the dose on the second day was 0.20 g/day. The intravenous dose of amphotericin B was 5.0 mg/day. On day 30, the patient had improved hemoptysis and cough symptoms, no other special discomfort, and stable vital signs after physical examination.

### 2.5. Whole-genome sequencing

In order to truly reflect the genome information of *A luchuensis* in clinical samples, whole-genome sequencing of pathogenic microorganisms was conducted directly on clinical samples. The complete, circular chromosome assembly of isolate T107D was 4629,767 bp long. After assembling the data, the genome of the strain was obtained (NGDC Accession Number: C_AA330775.1). The genome circle map is shown in Figure 1. The GC was 49.21% (Fig. 3A).

**Figure 2.**
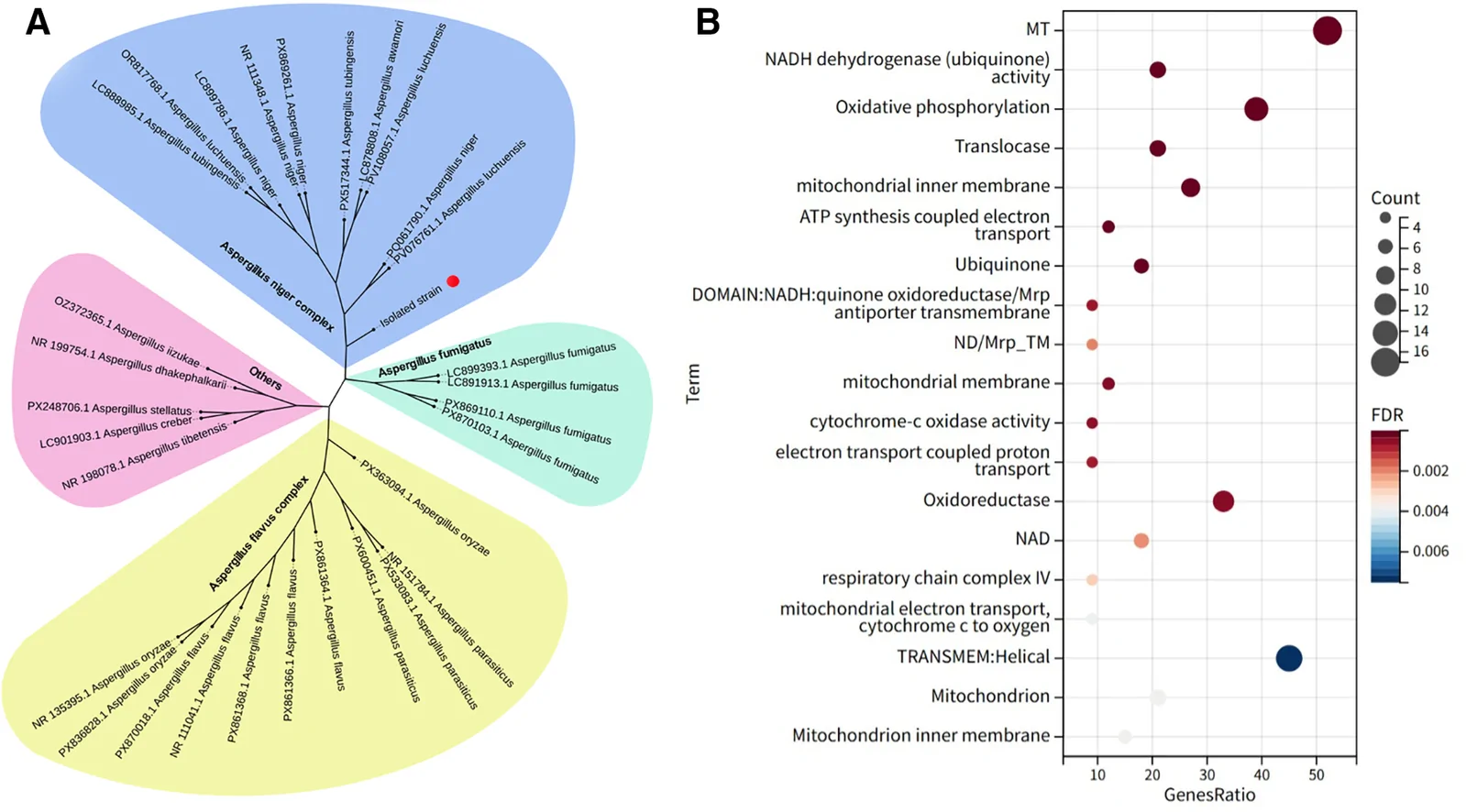
Species phylogenetic relationship and transcriptome functional-enrichment analysis of target genes. (A) Phylogenetic tree displaying the evolutionary classification and homology relationship of *Asp* species, including *A. fumigatus*, *A niger*, *A. flavus* and other related strains. The red dot marks the isolated-strain sequence. (B) Bubble plot for top-enriched Gene Ontology (GO) terms. The x-axis represents the gene ratio of enriched genes; the size of each bubble indicates the number of enriched genes (Count); the color gradient corresponds to the false-discovery rate (FDR), where darker-red dots stand for higher enrichment significance. Enriched terms are mainly clustered in mitochondrial inner-membrane, respiratory-chain, and oxidative-phosphorylation-related functions.

**Figure 3.**
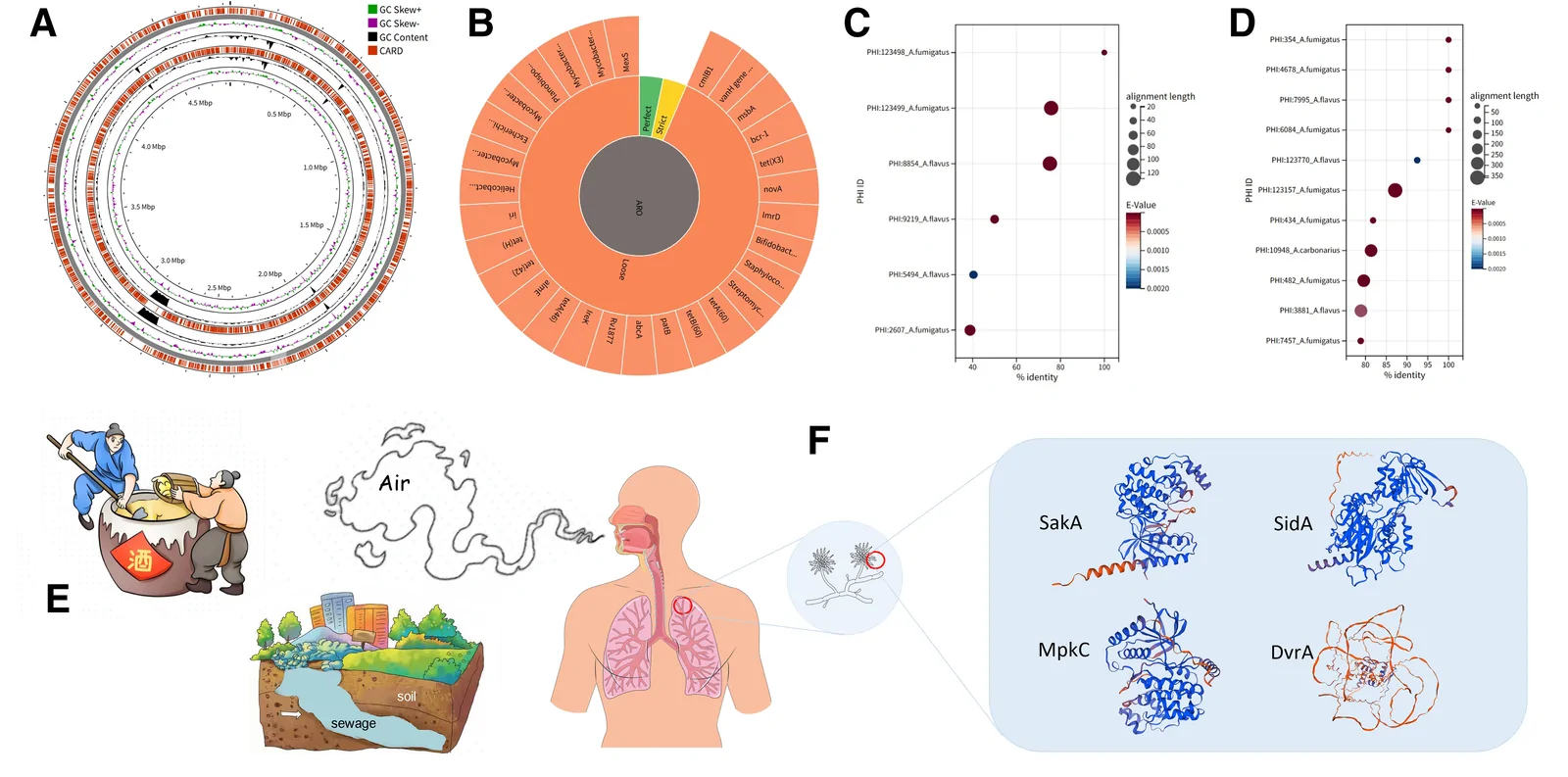
Infection and pathogenicity analysis of isolated *A luchuensis.* (A) Circular diagram of genome sequencing sequences; (B). Diagram of resistance genes analyzed via CARD; (C). Bubble chart of virulence factors (reduced virulence) in the isolated strains; (D). Bubble chart of virulence factors (increased virulence) in the isolated strain. (E). Potential routes of infection with the patient’s isolated strain. F. protein structure 3D modeling of hypervirulence factors via PHI-base.

### 2.6. Transcriptome sequencing

The transcriptome sequencing results show that 33 transcripts from the isolated strain were detected. Transcriptome Functional Enrichment Bubble Plot in Figure 2B. Enrichment analysis of the above transcripts revealed that chromosomal enrichment was concentrated in the mitochondria; in GO analysis, molecular functions were enriched in NADH dehydrogenase (ubiquinone) activity, cellular components were enriched in the mitochondrial inner membrane, biological processes were enriched in ATP synthesis coupled to electron transport, and signaling pathways were enriched in oxidative phosphorylation. Transcriptome enrichment results indicate that the mitochondrial energy metabolism of this strain is highly active within the patient.

### 2.7. Phylogenetic analysis

A phylogenetic tree based on sequencing and database data was used to describe the evolutionary relationships among species with Neighbor-Joining.^[6]^ The results show that this strain isolated sequence is close to *Asp* luchuensis strain and has evolutionary different from the *A fumigatus, A niger, A flavus* (Fig. 2A). Although the genetic distance indicates that the isolate belongs to the *A niger* complex, it still differs from the traditional *A luchuensis* type.

CARD (Comprehensive Antibiotic Resistance Database) searches for resistome in DNA sequences.^[7]^ The prediction results are shown in the Table 1 (Fig. 3B). The CRISPR prediction of the genome was performed by the CRISPRCasFinder software (v.4.2.20),^[8]^. A CRISPR sequence of 95bp in length was predicted (Repeat consensus/cas genes: 5′-AATGAGTT-GGATTGCGATAGAATAGCA-3′).

**Table 1:** Resistance gene analyzed via CARD.

ARO term	SNP	Detection criteria	AMR gene family	Drug class	Resistance mechanism	% Identity of matching region	% Length of reference sequence
msbA		Protein homolog model	ATP-binding cassette (ABC) antibiotic efflux pump	Nitroimidazole antibiotic	Antibiotic efflux	59	17.7
E coli EF-Tu mutants conferring resistaneto Pulvomycin	R234F	Protein variant model	Elfamycin resistant EF-Tu	Elfamycn antibiotic	Antibiotic target alteration	54.87	57.7
abcA		Protein homolog model	ATP-binding cassette (ABC) antibiotic efflux pump	Cephalosporin, penicillin Beta-lactam, peptide antibiotic, moenomycin antibiotic	Antibiotic efflux	54.26	32.02
abcA		Protein homolog model	ATP-binding cassette (ABC) antibiotic efflux pump	Cephalosporin, penicillin beta-lactam, peptide antibiotic, moenomycin antibiotic	Antibiotic efflux	53.93	13.5
iri		Protein homolog model	Rifampin monooxygenase	Rifamycn antibiotic	Antibiotic inactivation	53.85	49.27
novA		Protein homolog model	ATP-binding cassette (ABC) antibiotic efflux pump	Aminocoumarin antibiotic	Antibiotic efflux	51.88	111.22
Streptomyces venezuelae rox		Protein homolog model	Rifampin monooxygenase	Rifamycn antibiotic	Antibiotic inactivation	51.61	65.13
novA		Protein homolog model	ATP-binding cassette (ABC) antibiotic efflux pump	Aminocoumarin antibiotic	Antibiotic efflux	50.36	26.5
abcA		Protein homolog model	ATP-binding cassette (ABC) antibiotic efflux pump	Cephalosporin, penicillin beta-lactam, peptide antibiotic, moenomycin antibiotic	Antibiotic efflux	50.2	51.93
Streptomyces venezuelae rox		Protein homolog model	Rifampin monooxygenase	Rifamycin antibiotic	Antibiotic inactivation	50	26.89
abcA		Protein homolog model	ATP-binding cassette (ABC) antibiotic efflux pump	Cephalosporin, penicillin beta-lactam, peptide antibiotic, moenomycin antibiotic	Antibiotic efflux	50	30.65

Using diamond software, the amino acid sequence of the target species was compared with the PHI-base database, and the genes of the target species and their corresponding virulence factors were combined to obtain the annotation results.^[9]^ The results of the virulence factors annotated for this sample are shown in the Table 2. The PHI alignment resulted in 18 sequences of genes that play a role in the pathogen-host interaction, including 6 related sequences with hypervirulence, indicating a high virulence between the isolate and the host (Fig. 3C). Meanwhile, 11 sequences were identified with reduced virulence (Fig. 3D).

**Table 2:** The virulence factor of the isolated strain was compared with the PHI-base database.

Virulence factor strength	PHI ID	% Identity	Alignment length	Mismatches	E-Value	Bit score
Reduced_virulence	PHI:354_A.fumigatus	100	32	0	7.00E-171	65.1
	PHI:4678_A.fumigatus	100	32	0	7.00E-171	65.1
	PHI:7995_A.flavus	100	32	0	1.00E-169	65.1
	PHI:6084_A.fumigatus	100	19	0	2.00E-47	43.1
	PHI:123770_A.flavus	92.45	53	4	2.00E-22	107
	PHI:123157_A.fumigatus	87.15	358	45	0	670
	PHI:434_A.fumigatus	81.82	44	8	4.00E-151	57.4
	PHI:10948_A.carbonarius	81.34	284	53	2.00E-159	504
	PHI:482_A.fumigatus	79.58	289	59	2.00E-160	508
	PHI:3881_A.flavus	78.91	294	62	8.00E-161	509
	PHI:7457_A.fumigatus	78.85	52	11	1.00E-166	67.8
Increased_virulence (hypervirulence)	PHI:123498_A.fumigatus	100	19	0	2.00E-47	43.1
	PHI:123499_A.fumigatus	75.78	128	30	5.00E-87	218
	PHI:8854_A.flavus	75.19	129	31	9.00E-60	218
	PHI:9219_A.flavus	50	58	27	7.00E-09	64.3
	PHI:5494_A.flavus	40.38	52	29	0.002	46.6
	PHI:2607_A.fumigatus	38.82	85	48	2.00E-08	63.5
Loss of pathogenicity	PHI:486_A.fumigatus	81.44	501	91	0	816

Among the virulence factors defined in the appeal, 4 factors (PHI:486: SidA; PHI:123498: SakA; PHI:123499: MpkC; PHI:2607: DvrA) warrant particular attention. The above 4 virulence factors could confer the isolates with high invasiveness. To ensure the accuracy of the alignment results, 3D modeling was performed for each of the 4 virulence factors, and the results demonstrated that the active sites of all virulence factors were consistent (Fig. 3F).^[10]^ The active sites of the 4 factors are located in the center of the barrel structure.

## 3. Discussion and conclusions

In this case, the patient was non-neutropenic and presented with cough and hemoptysis without an obvious cause. The patient had opportunistic exposure to materials potentially containing *A luchuensis*, including sewage, sludge, fermentation starter, and others (Fig. 3E). CT scans revealed patchy opacities scattered in both lower lung lobes, suggesting possible inflammatory lesions. To rapidly identify the source of infection, metagenomic next-generation sequencing (mNGS) was performed on the patient’s sample. After 20 hours, *A luchuensis* (Reads: 20318) was detected. Fungal culture yielded negative results; fungal fluorescent staining was also negative, while the galactomannan assay (GM test) was positive, and *Asp* PCR returned a positive result. The negative results from conventional microbiology tests may be attributed to a low fungal load or growth requirements of this strain that differ from those of common pathogenic *Asp* species. With the continuous advancement and iteration of diagnostic technologies, molecular biology-based testing methods, including metagenomic next-generation sequencing (mNGS), are now providing critical support for the clinical diagnosis of fungal diseases due to their high sensitivity and rapid turnaround time. Simultaneously, these modern techniques complement conventional microbiological testing, thereby enhancing overall diagnostic capabilities and better serving clinical needs.^[11]^

*Aspergillus* is the leading cause of invasive mold infection and is a serious problem in immunocompromised populations worldwide. In the analysis of drug resistance genes through genome sequencing, although several bacterial resistance loci were identified, no strictly fungus-specific resistance-conferring mutations were detected. Analysis of virulence factors in the isolate using the PHI-base database revealed that this strain not only exhibits high virulence toward plants (PHI: 8854, PHI: 9219, PHI: 5494) but also harbors virulence factors associated with high pathogenicity in mammals (PHI:123498 SakA, PHI:123499 MpkC, PHI:2607 DvrA), demonstrating its strong invasiveness. *A fumigatus* germination in the airways is regulated by SskA through the SakA/MpkC mitogen-activated protein kinase (MAPK) pathway, and drives enhanced disease initiation and inflammation in the lungs.^[12]^ 11 reduced virulence factors were found, suggesting that the strain may also have weakened virulence. One virulence factor (PHI:486 SidA) was defined as loss of pathogenicity in the analysis. The survival of *Asp* in serum may be related to the secretion of siderophores. Many studies have indicated that sidA is necessary for *Asp* virulence.^[13,14]^ No fungal drug resistance genes were detected in the genomic sequence of this strain, which may explain the patient’s good recovery after antifungal medication.

*Aspergillus* is the primary cause of IPA, which remains associated with a mortality rate of 25% to 35% even with antifungal treatment.^[15]^ The disease is initiated by the inhalation of conidia, which deposit on the alveolar epithelial lining. In susceptible hosts, these adherent conidia then germinate, invade, and damage lung cells. The host cells respond by synthesizing proinflammatory mediators that recruit phagocytic white blood cells to the site of infection.^[16]^ Although these phagocytes may eliminate some of the invading fungi, they can also cause significant tissue destruction, thereby contributing to the pathogenesis of invasive aspergillosis. In this case, the strain-rarely associated with pulmonary fungal disease-caused symptoms such as cough and hemoptysis in a non-neutropenic patient, indicating its high invasiveness and underscoring the need for heightened clinical awareness. In the future, flow cytometry or chemiluminescence immunoassay (CLIA) analysis of fungal virulence gene antigens is expected to provide diagnostic evidence for invasive aspergillosis.^[17]^

## Author contributions

**Conceptualization:** Kai Chang.

**Funding acquisition:** Kai Chang, Xin Zhao.

**Visualization:** Kai Chang.

**Data curation:** Huajie Xie.

**Investigation:** Huajie Xie, Yanyan Wang, Ningwei Xian.

**Methodology:** Huajie Xie, Yanyan Wang, Xin Zhao, Wanlin Na, Ningwei Xian, Chenxia Liu.

**Formal analysis:** Yanyan Wang.

**Supervision:** Wanlin Na, Yuan Liu.

**Validation:** Wanlin Na, Zhongyong Jiang.

**Resources:** Yuan Liu, Zhongyong Jiang, Chenxia Liu.

**Software:** Chenxia Liu.

**Writing – original draft:** Kai Chang.

**Writing – review & editing:** Kai Chang, Yuan Liu.
